# Early gastric cancer detection and lesion segmentation based on deep learning and gastroscopic images

**DOI:** 10.1038/s41598-024-58361-8

**Published:** 2024-04-03

**Authors:** Kezhi Zhang, Haibao Wang, Yaru Cheng, Hongyan Liu, Qi Gong, Qian Zeng, Tao Zhang, Guoqiang Wei, Zhi Wei, Dong Chen

**Affiliations:** 1 Guangxi Key Laboratory of Information Functional Materials and Intelligent Information Processing, School of Physics and Electronics, Nanning Normal University, 175 Mingxiu East Road, Nanning, 530001 Guangxi China; 2 Department of Gastroenterology, Shandong Second Provincial General Hospital, 4 Duan Xing West Road, Jinan, 250022 Shandong China; 3 School of Electronic Engineering, Hunan College of Information, Changsha, 410200 Hunan China

**Keywords:** Deep learning, Early gastric cancer, Gastroscopic images, Mask R-CNN, Biomedical engineering, Gastric cancer, Gastric cancer, Oesophagogastroscopy

## Abstract

Gastric cancer is a highly prevalent disease that poses a serious threat to public health. In clinical practice, gastroscopy is frequently used by medical practitioners to screen for gastric cancer. However, the symptoms of gastric cancer at different stages of advancement vary significantly, particularly in the case of early gastric cancer (EGC). The manifestations of EGC are often indistinct, leading to a detection rate of less than 10%. In recent years, researchers have focused on leveraging deep learning algorithms to assist medical professionals in detecting EGC and thereby improve detection rates. To enhance the ability of deep learning to detect EGC and segment lesions in gastroscopic images, an Improved Mask R-CNN (IMR-CNN) model was proposed. This model incorporates a “Bi-directional feature extraction and fusion module” and a “Purification module for feature channel and space” based on the Mask R-CNN (MR-CNN). Our study includes a dataset of 1120 images of EGC for training and validation of the models. The experimental results indicate that the IMR-CNN model outperforms the original MR-CNN model, with Precision, Recall, Accuracy, Specificity and F1-Score values of 92.9%, 95.3%, 93.9%, 92.5% and 94.1%, respectively. Therefore, our proposed IMR-CNN model has superior detection and lesion segmentation capabilities and can effectively aid doctors in diagnosing EGC from gastroscopic images.

## Introduction

According to the International Agency for Research on Cancer, gastric cancer accounted for 5.6% of all new cancer cases and 7.7% of cancer deaths globally in 2020^1^. Despite a decline in the global incidence and mortality rates, the actual number of cases and deaths is still increasing, particularly in developing countries with aging populations^2,3^. Gastric cancer has a poor overall prognosis, with a 5-year survival rate of only 10%-30%, whereas patients with EGC have a survival rate of 70%-90%, highlighting the importance of early detection and treatment^4,5^. Although white-light endoscopy is the standard method for detecting EGC, its accuracy is heavily reliant on the expertise and experience of endoscopists, resulting in an accuracy rate of only 70–80%^6^. Furthermore, the large workload of medical image analysis also affects diagnostic results.

In recent years, artificial intelligence (AI) has been making remarkable progress in various fields, including healthcare^7^. Researchers are increasingly using AI techniques, such as computer vision methods based on deep learning, to assist in detecting EGC^8^. For example, Toshiaki Hirasawa's team^9^ developed a CNN diagnostic system that employs the Single Shot MultiBox Detector architecture to process endoscopic images quickly and accurately, achieving an overall sensitivity of 92.2% and a positive predictive value of 30.6%. Another study by Sakai et al.^10^ proposed a convolutional neural network scheme that employs transfer learning to automatically detect EGC in endoscopic images with an accuracy of 87.6%. These results demonstrate the potential of AI-assisted diagnosis to improve the efficiency and accuracy of clinical practice for endoscopists.

Recently, there has been a surge of interest in the application of AI technology in the medical field, and in particular, the use of computer vision methods for the EGC detection. Mitsuaki Ishioka et al.^11^ developed a CNN system for detecting and recognizing gastric cancer in video images with an impressive accuracy of 94.1%. Similarly, Yoon et al.^12^ developed an optimized model for EGC detection and prediction, which achieved high sensitivity (91%) and overall AUC (98.1%). Hiroya Ueyama et al.^13^ constructed an AI-based diagnosis system, "CNN-CAD", for detecting EGC in ME-NBI images, which demonstrated an overall accuracy, sensitivity, and specificity of 98.7%, 98%, and 100%, respectively. Zhigang Song et al.^14^ developed a clinical application system that achieved near-perfect sensitivity and an average specificity of 80.6% on a self-built dataset, suggesting that the system can help specialists improve diagnostic efficiency and prevent misdiagnosis. Hirotaka Oura et al. ^15^ developed a Double-Check Support System (DCSS) for esophagogastroduodenoscopy (EGD) still images, which outperformed endoscopists with an accuracy, sensitivity, and positive predictive value of 83.3%, 89.2%, and 93.3%, respectively. Lianlian Wu et al.^16^ developed the ENDOAN-GEL system, which demonstrated an average accuracy, sensitivity, and specificity of 84.7%, 100%, and 84.3%, respectively, for detecting gastric cancer and improving the quality of EGD. Finally, Shibata et al.^17^ investigated EGC detection and lesion segmentation using MR-CNN, achieving high sensitivity (96.0%) and low false positives (0.10 FP/image) per image. These findings suggest that AI-based detection systems have the potential to significantly improve the accuracy and efficiency of EGC detection and diagnosis.

After analyzing recent research on EGC detection based on deep learning, it has been found that the object detection algorithm is mainly used due to its advantages such as small size, high efficiency, and fast detection. However, the detection results only provide a rectangular bounding box in the lesion region, which covers both the lesion and non-lesion regions. This approach cannot accurately indicate the infiltrated region of gastric cancer, which is unfavorable to physicians for making accurate diagnoses. Our research objective is to improve the accuracy of EGC detection and lesion segmentation in gastroscopic images using a deep learning model. Since the clinical features of EGC are not always apparent, we had employed the classical object segmentation algorithm, MR-CNN^18^, based on which IMR-CNN was formed by adding a “Bi-directional feature extraction and fusion module” and a “Purification module for feature channel and space” to be better suited for detecting EGC. Our IMR-CNN model not only achieves precise detection of EGC but also performs high-precision segmentation of lesions. This indicates that our method is capable of detecting EGC while also providing additional information on the EGC lesions. The validation of our model was conducted on a self-built dataset and the publicly available Kvasir polyp segmentation dataset, and the IMR-CNN showed significant improvement over MR-CNN in terms of the primary evaluation metrics.

## Materials and method

### Deep‑learning models

**Figure 1:**
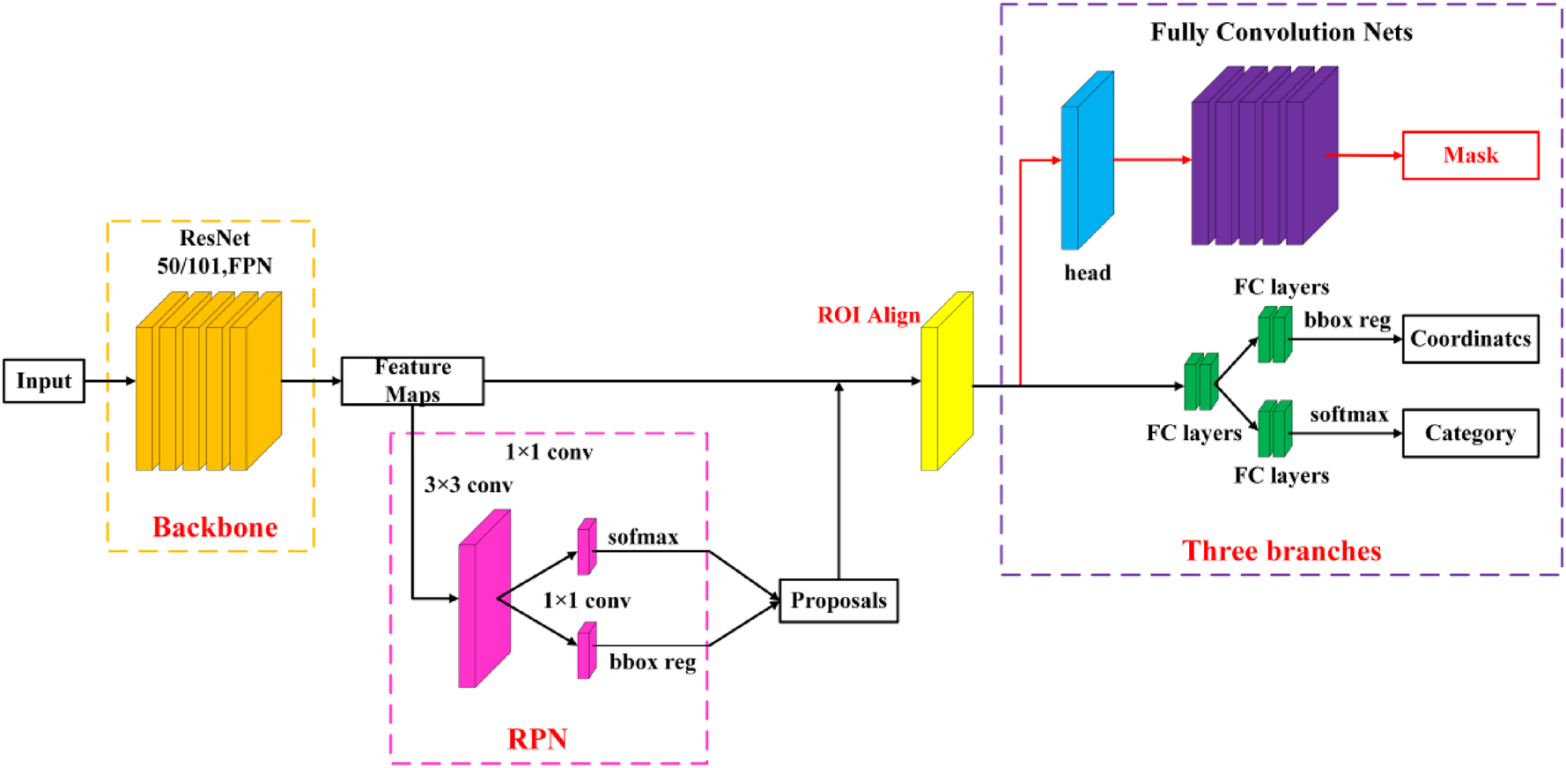
The overall structure of MR-CNN.

MR-CNN is a simple and efficient instance segmentation network proposed by Kaiming He et al.^18^. Based on Faster R-CNN^19^, a new segmentation branch in parallel is added to achieve object detection and instance segmentation. The overall structure of MR-CNN can be divided into three parts: (i) a convolutional backbone for feature extracting of the input image, (ii) a region proposal network (RPN) for generating interesting regions, and (iii) a network head with three branches for classification, bounding box regression, and segmentation mask. The structure of MR-CNN is demonstrated in Fig. 1.

**Figure 2:**
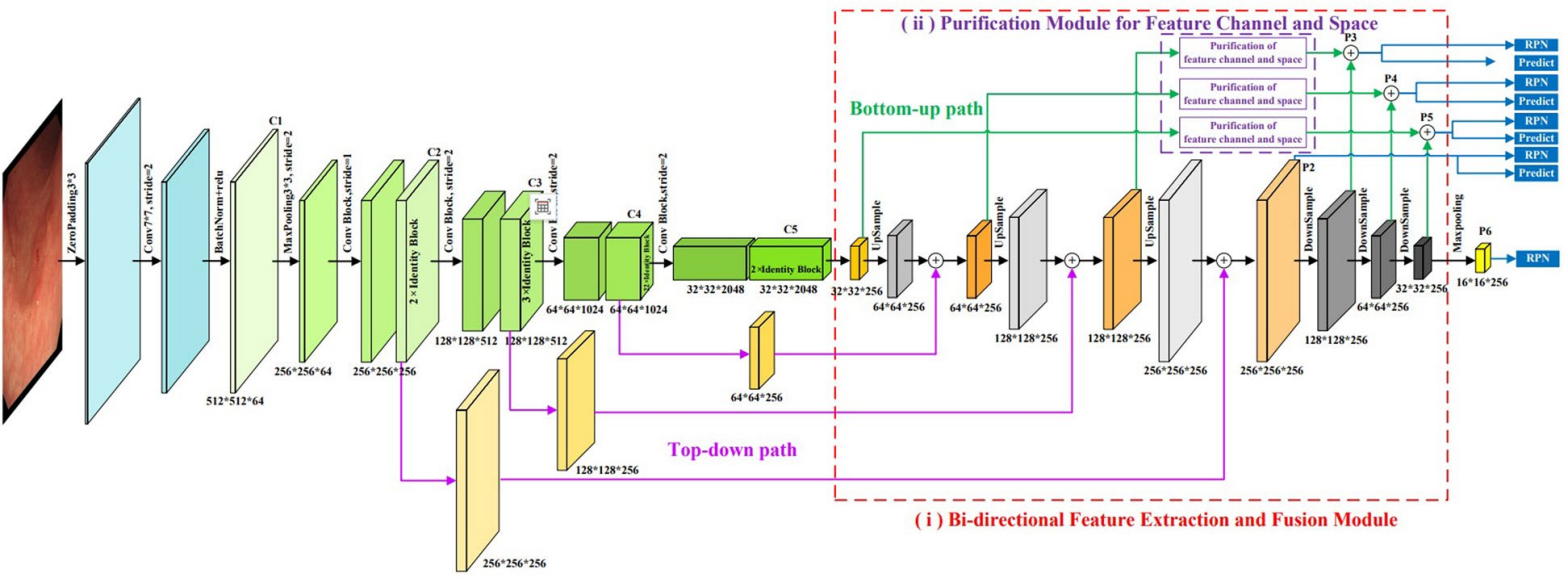
The backbone network of IMR-CNN.

In this paper, the convolutional backbone network of MR-CNN was improved to enhance the capability of EGC feature extraction in gastroscopic images, and then high-quality results for object detection and segmentation were obtained. The improvement included two aspects: (i) a Bi-directional Feature Extraction and Fusion Module; (ii) a Purification Module for Feature Channel and Space. The structure of the improved convolutional backbone network is shown in Fig. 2. To verify the interaction of the two modules, another model was formed by adding only the “Bi-directional Feature Extraction and Fusion Module”, that is named IBMR-CNN.

#### Bi-directional feature extraction and fusion module

Generally, when image features are extracted by CNN, the low-level feature maps rich in spatial information and little semantic information, while the high-level feature maps are just the opposite. Spatial information and semantic information are helpful for object location and object classification, respectively. So, the appropriate use of the two types of information is beneficial for improving the accuracy of object detection and segmentation. The Feature Pyramid Network (FPN)^20^ was introduced into the backbone network of MR-CNN. The multi-scale feature map is extracted by the bottom-up CNN, while the high-level features and low-level features are fused layer by layer through the top-down FPN structure and horizontal connectivity. Then a multi-scale feature map with semantic information and spatial information fusion is generated, which enhances the model’s ability to detect small objects.

To enhance the accuracy of EGC detection in gastroscopic images, the FPN structure is improved furtherly. A bottom-up feature fusion path has been added based on the FPN structure by referring to the Path Aggregation Network (PANet)^21^. The bottom-up feature fusion path allows the spatial information of low-level features to be better fused into the semantic information of high-level features. So that, the feature extraction capability of the model is improved. The module is displayed in the red dotted line box in Fig. 2.

#### Purification module for feature channel and space

**Figure 3:**
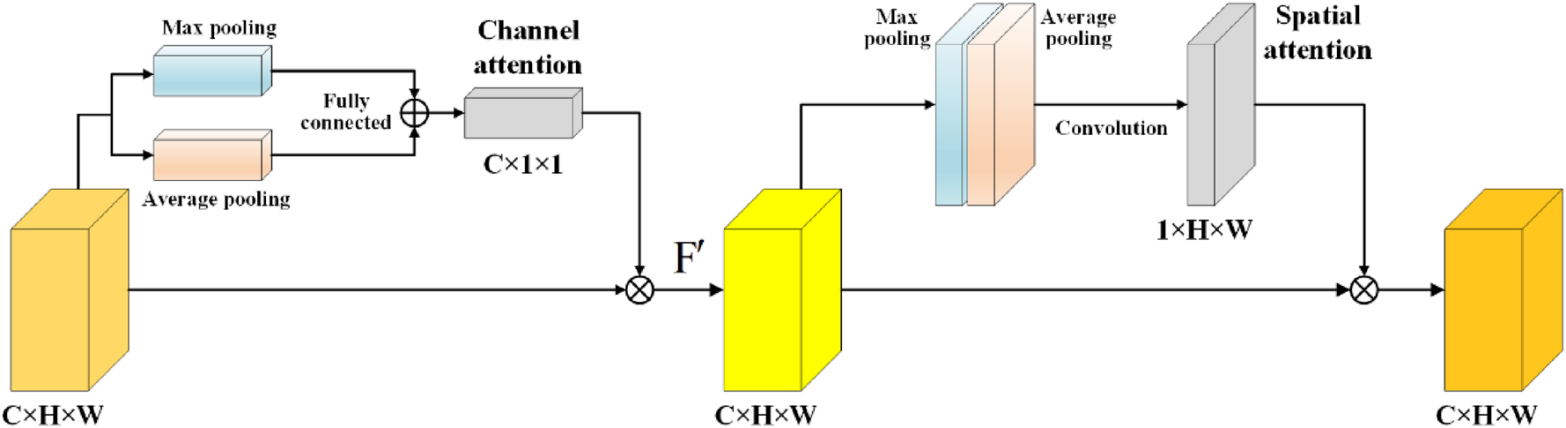
The structure of “Purification module for feature channel and space”.

It is well known that attention mechanism focuses on important features and suppressing unnecessary ones to obtain valuable information as efficiently as possible in limited computing resources^22^. The attention mechanism can be classified into channel attention, spatial attention, and hybrid attention. The channel attention focuses on the key features of the object, such as SENet^23^, BAM^24^, etc. The spatial attention pays more attention to the key location of the object, such as STNet^25^, DCNet^26^, OPAM^27^, etc. The hybrid attention combines the capabilities of the two attentions, such as CBAM^22^, DANet^28^, Coordinate Attention^29^, etc. Considering our research objective, we should pay attention to not only the key features of the channel attention but also the key positions of the spatial attention. Therefore, the idea of CBAM was used to improve our model. As displayed in the purple dashed box in Fig. 2, an attention mechanism is introduced before the fusion of feature layers from different paths, aiming to enhance the features related to the object. We refer to this as “Purification module for feature channel and space”, and its structure is shown in Fig. 3. The following describes the details of the attention. The input feature map from each channel is max-pooled and average-pooled, generating two 1D vectors, which are sent to the fully connected layer and added to generate a 1D channel attention map. The channel attention map is then multiplied by the input feature map, yielding a channel attention-adjusted feature map F'. Then the F' map is max-pooled and average-pooled along the channel axis, generating two 2D maps. The F’ maps are then concatenated and convolved by a standard convolution layer, producing a 2D spatial attention map. The 2D spatial map is multiplied with the F' map element-wise. Finally, the feature map adjusted by spatial attention and channel attention with multi-scales can be obtained.

### Evaluation metrics

To evaluate the performance of models quantitatively and objectively, some evaluation metrics^30,31^ need to be introduced. According to the research, we reasonably set the statistical data^32^ and evaluation metrics for the experiment, as listed in Tables 1 and 2, respectively.

**Table 1:** Confusion matrix of experimental.

	Predicted value	
	Positive sample	Negative sample
Actual value		
Positive sample	True positive (TP. The EGC lesion region was correctly recognized)	False negative (FN. The EGC lesion region was not correctly recognized)
Negative sample	False positive (FP. The normal region was recognized as the EGC lesion)	True negative (TN. The normal region was not recognized as the EGC lesion)

**Table 2:** Evaluation metrics.

Evaluation metrics	Calculation formula	Evaluation meaning
Precision	${\text{Precision}} = \frac{TP}{{TP + FP}}$	The percentage of all predictive positive samples correctly recognized as positive
Recall/sensitivity	${\text{Recall}} = \frac{TP}{{TP + FN}}$	The percentage of all actual positive samples that are correctly recognized as positive
Specificity	${\text{Specificity}} = \frac{TN}{{TN + FP}}$	The percentage of all actual negative samples correctly recognized as negative
Accuracy	${\text{Accuracy}} = \frac{TP + TN}{{TP + FP + TN + FN}}$	The percentage of samples with correct recognition results among all samples
F1 score	$F1{\text{ s}}core = \frac{{{2} \times TP}}{2 \times TP + FP + FN}$	A measure of a test’s accuracy by calculating the harmonic mean of the precision and recall
Intersection over union (IoU)	$IoU = \frac{A \cap B}{{A \cup B}}$	Predicted bounding box overlap with real bounding box
Average precision (AP)	None	The average of per-class precision
Precision-recall curves (PR curve)	None	Relationship curves of Precision and Recall under different thresholds

### Datasets

In the work, two datasets were prepared for experiments: a self-built EGC dataset and a public polyp dataset. Due to lack of EGC public dataset, the public Kvasir-SEG^33^ polyp dataset was selected solely for segmentation task. To compare the performance of the three models, they were trained and tested on the both datasets. Although the two datasets differ significantly, they have little impact on the performance of models and help to verify the generalization performance of the models. We confirm that all methods were performed in accordance with the relevant guidelines and regulations, which include the Declaration of Helsinki, the International Ethical Guidelines for Human Biomedical Research (International Committee for the Organization of Medical Sciences), and the Guidelines for the Construction of Clinical Research Ethics Review Committees Involving Human Beings (National Health Commission of the People’s Republic of China, 2019 Edition).

#### The self-built EGC dataset

In the experiment, a total of 1,120 images were collected from 26 healthy subjects (140 images) and 180 cases (980 images) of gastric cancer for preoperative examinations between January 2020 to December 2021 in Shandong Second Provincial General Hospital. The size of images is 1080 × 1080 pixels. We were authorized to obtain gastroscopic images anonymously, and this research was approved by the Ethics Committee of the Shandong Second Provincial General Hospital. The number of Ethics Review Approval Statement is 20220703.

The gastroscopic images were analyzed using a multi-view k-proximal plane clustering algorithm^34^ and verified to be valid data. Then, these images were strictly marked according to the recommendations of professional gastroscopists, and the marked images were verified by professional physicians. The 1120 images were randomly divided into training dataset, validation dataset and testing dataset with the ratio of 12:3:5.

#### The public Kvasir-SEG dataset

The Kvasir-SEG dataset^33^ (available from https://datasets.simula.no/kvasir-seg/) is an extension of the Kvasir dataset^32^, which is the first multiclass dataset based on gastrointestinal disease. The Kvasir-SEG dataset contains 1000 annotated polyp images and their mask images for segmentation. According to the mask images, the annotated polyp images were re-marked strictly and new mask images were generated to meet experimental requirements. Similarly with self-built dataset, the Kvasir-SEG dataset was randomly divided into training dataset, validation dataset and testing dataset with the ratio of 12:3:5.

### Training and testing

In this work, MR-CNN, IMR-CNN, and IBMR-CNN models were trained and tested on the two datasets to clearly demonstrate the ability of the improved model to detect EGC and segment lesion.

To begin with, the three models were trained on the self-built dataset by using migration learning, respectively. COCO pre-trained weights were loaded and the learning rate was set to 1.0e−5, the weight attenuation value was set to 1.0e−8, the threshold value was set to 0.7, and the number of training iterations was set to 100. The other training parameters were kept consistent. The optimal weights for each model were selected via a comprehensive evaluation based on several major metrics, such as Precision, Recall, Specificity and F1-Score. And then, the models were tested on the test dataset of the self-built dataset. The performance of the models was evaluated via the test results.

Furthermore, to verify the robustness and reliability of the three models, a fivefold cross-validation method was employed^17^ on the self-built EGC dataset.

Finally, the three models were trained on the public dataset with the same training method and parameter settings as on the self-built dataset, and then tested on the test dataset of the public dataset. The purpose is to verify the generalization performance of the models and illustrate the superior performance of our model objectively.

### Ethics approval and consent to participate

This research was approved by the Ethics Committee of the Shandong Second Provincial General Hospital, and the Ethics Committee waived the requirement for informed consent of the subjects. The number of Ethics Review Approval Statement is 20220703.

## Results and discussion

**Figure 4:**
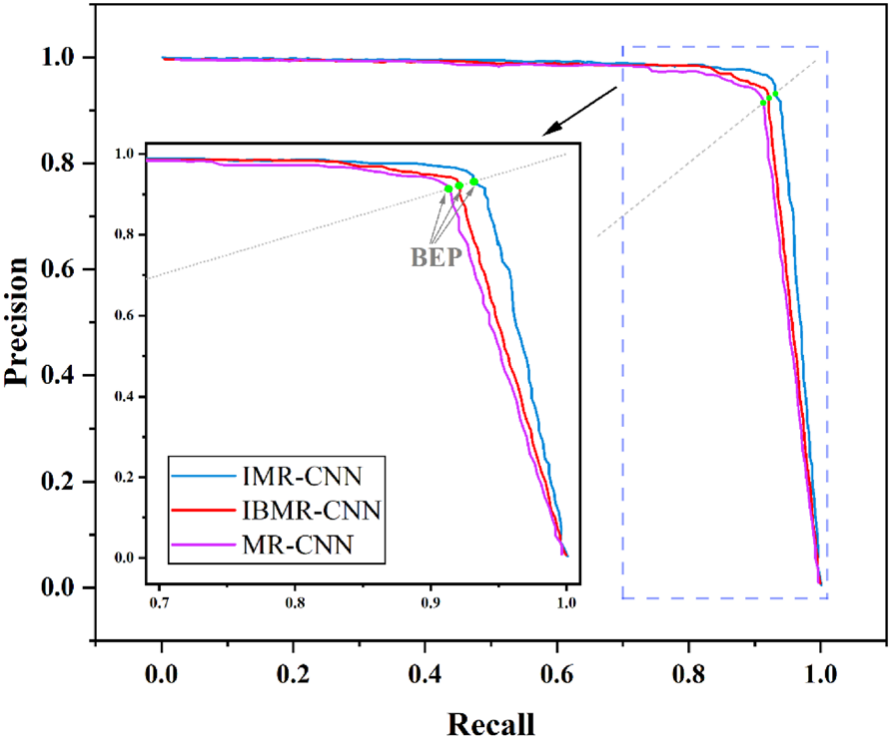
The PR curves of three models tested on the self-built dataset.

The three models were tested on the self-built dataset, and obtaining the PR curves, AP values and IoU values, which are summarized in Fig. 4 and Table 3. Break-Even-Point (BEP) on the PR curve is a valuable evaluation metric in which Precision and Recall are considered comprehensively, and it is the value when Precision and Recall are equal. As shown in Fig. 4, the BEP value of IMR-CNN is larger than that of MR-CNN and IBMR-CNN. As displayed in Table 3, compared with MR-CNN, the AP and IoU values of IMR-CNN increase by 2.8% and 0.8%, and those of IBMR-CNN increased by 0.8% and 0.5%, respectively. The results demonstrate that IMR-CNN exhibits superior accuracy compared to MR-CNN in both EGC detection and lesion segmentation. To enhance the robustness of the findings, a rigorous fivefold cross-validation was performed on a self-built dataset, verifying the reliability of the models. The corresponding values of AP and IoU were recorded in each experiment. The average values are taken as the result of the experiment, as listed in Table 4. The AP and IoU of IMR-CNN are higher than those of IBMR-CNN and MR-CNN in EGC detection and lesion segmentation.

**Table 3:** The values of AP and IoU for different models.

Models	The self-built EGC dataset		The public Kvasir-SEG dataset	
	AP (%)	IoU (%)	AP (%)	IoU (%)
MR-CNN	90.7	78.3	89.8	83.2
IBMR-CNN	91.5	78.8	90.7	84.5
IMR-CNN	93.5	79.1	91.6	84.6

**Table 4:** The results of fivefold cross-validation on the self-built dataset.

Training time	MR-CNN		IBMR-CNN		IMR-CNN	
	AP (%)	IoU (%)	AP (%)	IoU (%)	AP (%)	IoU (%)
1	91.2	78.2	91.3	79.3	93.7	79.5
2	90.6	78.5	90.8	78.4	93.5	78.9
3	90.3	78.1	91.6	79.0	93.1	78.8
4	90.8	78.7	92.0	78.7	94.2	78.6
5	89.9	78.6	91.8	78.8	92.9	79.2
Average ± standard deviation	90.6 ± 0.4	78.4 ± 0.2	91.5 ± 0.4	78.8 ± 0.3	93.5 ± 0.5	79.0 ± 0.3

**Figure 5:**
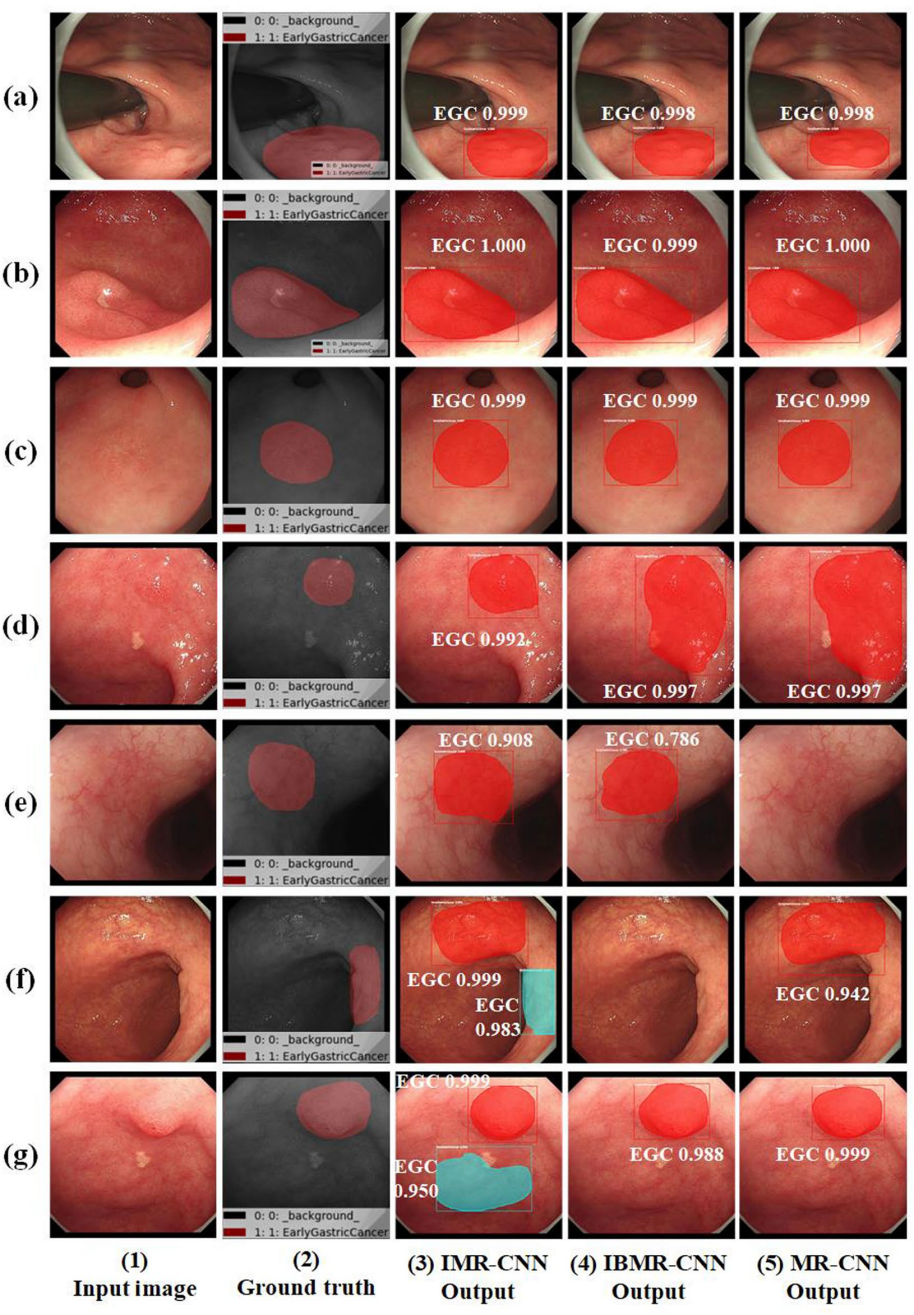
Examples of test results on the self-built dataset for the three models: (**a**–**c**) EGC detection and lesion segmentation were successfully performed; (**d**) there was/were FP for IBMR-CNN and MR-CNN; (**e**) FN for MR-CNN; (**f**) FP for IMR-CNN, FN for IBMR-CNN, FN and FP for MR-CNN; (**g**) FP for IMR-CNN.

The examples of the test results on the test set of self-built dataset are presented in Fig. 5. The EGC detection results on the test set of self-built dataset are listed in Table 5. The corresponding values of TP, TN, FP, and FN were counted for each model, and Precision, Recall, accuracy, specificity, and F1-scores were calculated. By comparing the evaluation metrics of the three models in Table 5, the values of all metrics of IMR-CNN are higher than those of IBMR-CNN and MR-CNN. The values of Precision, Recall, Accuracy, Specificity and F1-Score for IMR-CNN are 92.9%, 95.3%, 93.9%, 92.5% and 94.1%, respectively. These results indicate that the IMR-CNN model has better performance in EGC detection and lesion segmentation from gastroscopic images. This enhanced performance of IMR-CNN can be attributed to two primary factors. First, the model has been enhanced in feature learning and precise positioning of the target object by the bi-directional feature extraction and fusion module. Second, the model purifies the irrelevant features and strengthens the relevant features via the feature channel and spatial purification mechanism.

**Table 5:** The test results of EGC detection on the self-built dataset.

	TP	TN	FP	FN	Precision (%)	Recall (%)	Accuracy (%)	Specificity (%)	F1 (%)
MR-CNN	251	229	30	21	89.3	92.3	90.4	88.4	90.8
IBMR-CNN	258	239	24	17	91.5	93.8	92.3	90.9	92.6
IMR-CNN	263	247	20	13	92.9	95.3	93.9	92.5	94.1

**Figure 6:**
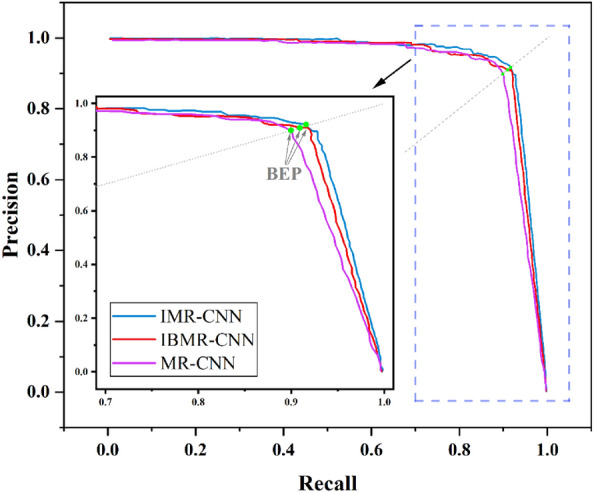
The PR curves of the three models tested on the public dataset.

The three models were also trained and tested on the public dataset, and the test results are shown in Fig. 6 and Table 3. Analyzing the PR curves of the three models, it is evident that IMR-CNN achieves the highest BEP value, further substantiating its superior performance over MR-CNN. In Table 3, the AP and IoU values of IMR-CNN are increased compared with MR-CNN, which also indicates that the detection and segmentation ability of IMR-CNN has been improved. In summary, IMR-CNN not only improves the accuracy of EGC detection and lesion segmentation in gastroscopic images, but also suitable for polyp detection in gastroscopic images. That means that IMR-CNN improves the performance and has better generalization performance compared with MR-CNN.

## Conclusion

In this paper, we present an enhanced IMR-CNN model, which builds upon the foundation of the MR-CNN architecture by incorporating two novel modules: the "Bi-directional feature extraction and fusion module" and the "Purification module for feature channel and space". The experimental results demonstrate that the IMR-CNN model is higher than the original MR-CNN model in several major evaluation metrics, such as Precision, Recall, Specificity and F1-Score. The IMR-CNN model could be used to detect EGC and segment the lesion from gastroscopic images accurately, so as to help doctors to diagnose the disease better. This will help to improve the detection rate of EGC and reduce the risk of missed and false detections.

In future work, some new methods, such as graph learning^35^ and multi-view clustering algorithms^36^, will be introduced into the model to obtain richer image information, and then the whole performance of the model will be improved. Assisted medical diagnosis is an important research field, so we will continue to monitor its development and further explore the application of deep learning in EGC detection.

## Data Availability

The public Kvasir-SEG dataset could be downloaded from https://datasets.simula.no/kvasir-seg/. The self-built EGC dataset used and/or analyzed during the current study is available from the corresponding author on reasonable request.
