# Author Correction: Early gastric cancer detection and lesion segmentation based on deep learning and gastroscopic images

**DOI:** 10.1038/s41598-024-59794-x

**Published:** 2024-04-19

**Authors:** Kezhi Zhang, Haibao Wang, Yaru Cheng, Hongyan Liu, Qi Gong, Qian Zeng, Tao Zhang, Guoqiang Wei, Zhi Wei, Dong Chen

**Affiliations:** 1 Guangxi Key Laboratory of Information Functional Materials and Intelligent Information Processing, School of Physics and Electronics, Nanning Normal University, 175 Mingxiu East Road, Nanning, 530001 Guangxi China; 2 Department of Gastroenterology, Shandong Second Provincial General Hospital, 4 Duan Xing West Road, Jinan, 250022 Shandong China; 3 School of Electronic Engineering, Hunan College of Information, Changsha, 410200 Hunan China

Correction to: *Scientific Reports* 10.1038/s41598-024-58361-8, published online 03 April 2024

The Acknowledgements section in the original version of this Article was incomplete.

“This research was supported by Guangxi Science and Technology Department|Specific Research Project of Guangxi for Research Bases and Talents (No. AD22080022), Medical and Health Science and Technology Development Project of Shandong (No. 202103030876), National Natural Science Foundation of China (No. 62161031), Guangxi Natural Science Foundation (No. 2020GXNSFBA297097), and Fund of Shandong Second Provincial General Hospital (No. 2023MS10).”

now reads:

“This research was supported by Guangxi Science and Technology Department | Specific Research Project of Guangxi for Research Bases and Talents (No. AD22080022), Project for Enhancing Young and Middle-aged Teacher's Research Basis Ability in Colleges of Guangxi (No. 2020KY09028), Medical and Health Science and Technology Development Project of Shandong (No. 202103030876), National Natural Science Foundation of China (No. 62161031), Guangxi Natural Science Foundation (No. 2020GXNSFBA297097), and Fund of Shandong Second Provincial General Hospital (No. 2023MS10). We are grateful to Enshuang Gao of Nanning Normal University for collecting and assembling of data.”

The original Article has been corrected.

